# Explainable AI Approaches in Federated Learning: Systematic Review

**DOI:** 10.2196/69985

**Published:** 2026-02-03

**Authors:** Titus Tunduny, Bernard Shibwabo

**Affiliations:** 1 School of Computing & Engineering Sciences Strathmore University Nairobi Kenya

**Keywords:** explainable artificial intelligence, federated learning, explainable federated artificial intelligence, privacy-preserving machine learning, model interpretability

## Abstract

**Background:**

Artificial intelligence (AI) has, in the recent past, experienced a rebirth with the growth of generative AI systems such as ChatGPT and Bard. These systems are trained with billions of parameters and have enabled widespread accessibility and understanding of AI among different user groups. Widespread adoption of AI has led to the need for understanding how machine learning (ML) models operate to build trust in them. An understanding of how these models generate their results remains a huge challenge that explainable AI seeks to solve. Federated learning (FL) grew out of the need to have privacy-preserving AI by having ML models that are decentralized but still share model parameters with a global model.

**Objective:**

This study sought to examine the extent of development of the explainable AI field within the FL environment in relation to the main contributions made, the types of FL, the sectors it is applied to, the models used, the methods applied by each study, and the databases from which sources are obtained.

**Methods:**

A systematic search in 8 electronic databases, namely, Web of Science Core Collection, Scopus, PubMed, ACM Digital Library, IEEE Xplore, Mendeley, BASE, and Google Scholar, was undertaken.

**Results:**

A review of 26 studies revealed that research on explainable FL is steadily growing despite being concentrated in Europe and Asia. The key determinants of FL use were data privacy and limited training data. Horizontal FL remains the preferred approach for federated ML, whereas post hoc explainability techniques were preferred.

**Conclusions:**

There is potential for development of novel approaches and improvement of existing approaches in the explainable FL field, especially for critical areas.

**Trial Registration:**

OSF Registries 10.17605/OSF.IO/Y85WA; https://osf.io/y85wa

## Introduction

### Background

Machine learning (ML) has become increasingly prevalent in critical sectors such as health care and security [1,2] driven by the need to process copious amounts of edge device data [3]. However, highly performant ML algorithms often operate as “black boxes” [4,5], creating a need for ML explainability to build trust. This has led to increased research in the field of explainable artificial intelligence (XAI) [2,4,6]. How a ML model works is important in building trust and reliability in its prediction or classification results, especially in critical areas. XAI approaches such as linear interpretable model-agnostic explanations (LIME) [7] and Shapley Additive Explanations (SHAP) [8] perform well with centralized models, although challenges remain [9]. Growing data privacy legislation such as the General Data Protection Regulation [10], HIPAA (Health Insurance Portability and Accountability Act) [11], and Kenya’s Data Protection Act [12] have further complicated centralized ML development.

**Figure 1:**
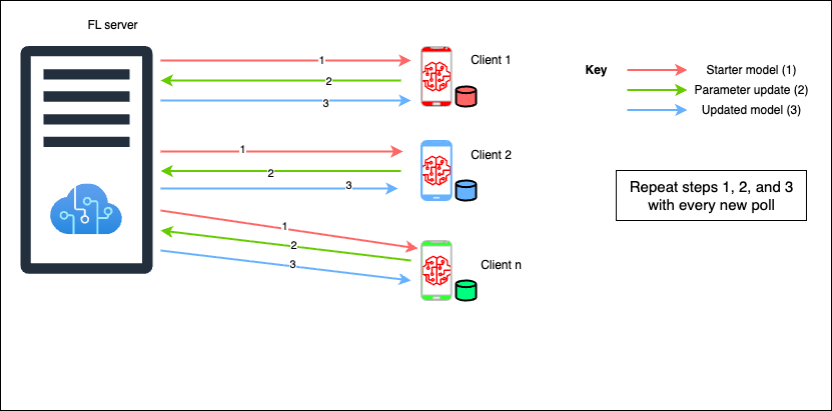
Federated machine learning process showing global model distribution and update of the global model on the federated learning (FL) aggregation server.

Federated learning (FL), introduced by McMahan et al [13] in 2016, enables privacy-preserving training on decentralized data stored on edge devices [13,14]. A central server distributes a global model to clients, who train it locally and send updates (learned parameters) back, ensuring that data never leave the device. The federated ML process is outlined in Figure 1. These updates are aggregated from selected clients (polling) typically using the federated average algorithm [13] to refine the global model. This process is repeated over several rounds, preserving privacy while improving model performance [15]. The federated averaging algorithm is outlined in Textbox 1.

**Federated averaging algorithm showing its mechanism.**

**Instructions**

Initialize global model weights *w*_0_

For each communication round *t*= 1, 2,..., *T* do

Server sends current model weights *w_t_* to a subset of clients

  Each selected client *k* trains on local data for *E* epochs with learning rate *η*:

  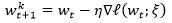, where ξ is a batch of local data

  Clients send updated weights *w*_*t+1*_^*k*^ back to the server

  Server aggregates client updates:

  
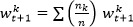
 (weighted by client data size)

End For

Return final global model weights w*

FL has demonstrated its potential as a privacy-preserving technique suitable for real-world applications despite its challenges [16,17]. However, its deployment in sensitive domains such as patient-embedded devices requires a high level of trust. This opens up significant research opportunities in integrating XAI techniques in FL environments. By enabling explanations on model generalizations at the data source while maintaining privacy, XAI can offer real-time benefits and enhance trust in artificial intelligence (AI)–driven embedded systems. FL can be categorized based on communication architecture or data partitioning. By communication architecture, FL models can be categorized as centralized or decentralized. By data partitioning, FL models can be categorized as horizontal, vertical, or transfer learning (TL) [18].

### Centralized FL

**Figure 2:**
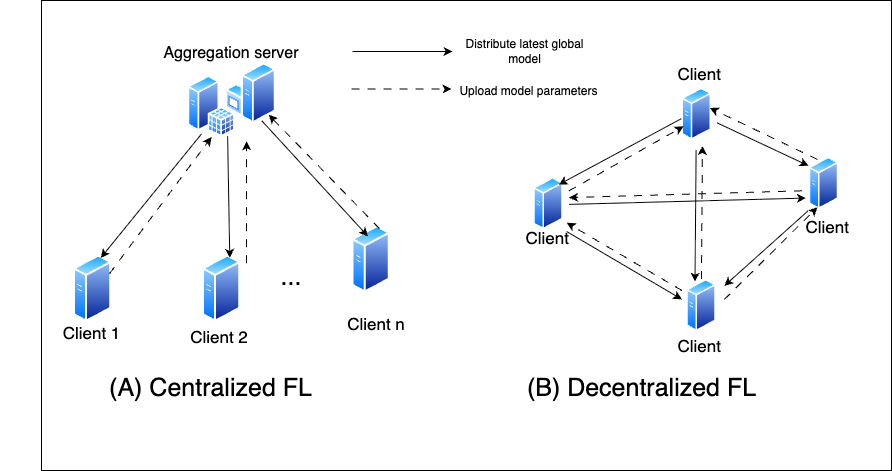
Centralized and decentralized federated learning (FL) in action.

In centralized FL (CFL), a global model is shared with various clients, who train it locally and send back the learned parameters. The server aggregates these updated parameters using algorithms such as federated averaging to improve the global model. Clients are selected through polling, and differential privacy can be applied by adding noise to the updates. CFL faces challenges such as client heterogeneity, limited communication and computing resources, fairness, security, and trust [19]. The structure of CFL is shown in Figure 2A.

### Decentralized FL

Decentralized FL—also known as distributed FL—eliminates the need for a central server. Each client trains a local model and shares the parameters with their peers using protocols such as pointing, gossip, and broadcast. Clients act as both learners and aggregators while refining their model based on peer updates. Therefore, the global model is developed from peer to peer [20,21]. The structure of decentralized FL is shown in Figure 2B.

### Horizontal FL

**Figure 3:**
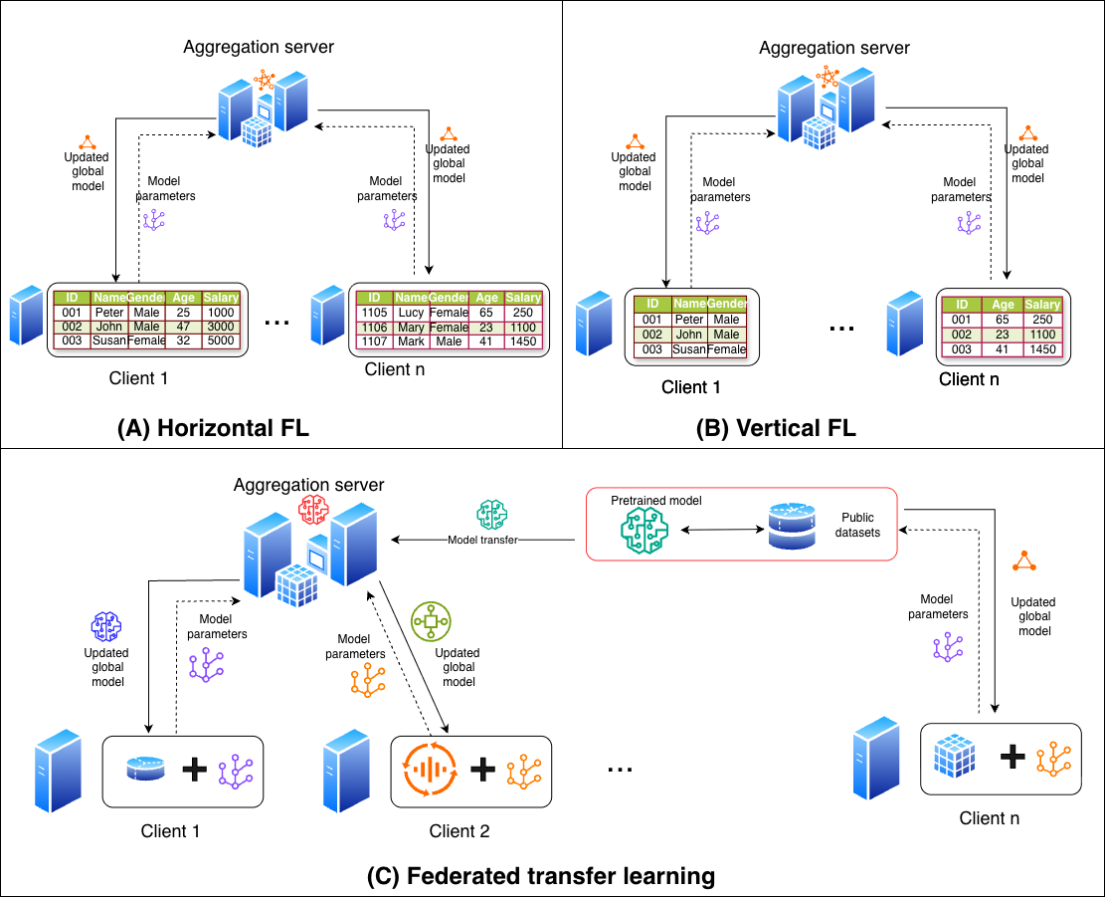
Federated learning (FL) types, showing horizontal FL, vertical FL, and federated transfer learning.

Horizontal FL (HFL) involves clients that share the same data features but have different data samples. Each client holds instances with similar attributes (eg, name, gender, date of birth, and salary), but the individual records (samples and rows) differ. This setup is ideal when datasets have high feature overlap across clients but differ in the entities they contain [22]. Figure 3A depicts the structure of HFL.

### Vertical FL

Vertical FL (VFL) is where clients share the same data samples but have different feature sets. Each client holds part of the information for the same users; for example, one client may have demographic data, whereas another may have financial data. VFL is ideal when full data sharing is not possible, such as in health care settings with multiple institutions holding complementary patient data [23]. Figure 3B shows the structure of VFL.

### Federated TL

Federated TL (FTL) merges the concepts of FL and TL. In FTL, a pretrained model from a related task is distributed to all the clients. Each client fine-tunes (adapts) the pretrained model using their local data. FTL is useful when training data are limited or privacy sensitive, such as in health care, allowing clients to benefit from existing models while preserving data privacy. FTL structured is showcased in Figure 3C.

### Contributions

This study makes contributions to the field of explainable FL in the following ways: it offers original insights into the explainability of FL models, including the methods used to explain the models, whether novel or existing, and how they have been used. This study also delves into the deployment contexts for FL models, including the types of FL used. Unlike prior works such as the study by Singh et al [24], which broadly examines FL applications, and the study by Aggarwal et al [25], which explores general FL use cases, this study also focused on the application areas for explainable FL models and their associated challenges, as well as providing the direction of the trends.

## Methods

### Overview

**Figure 4:**
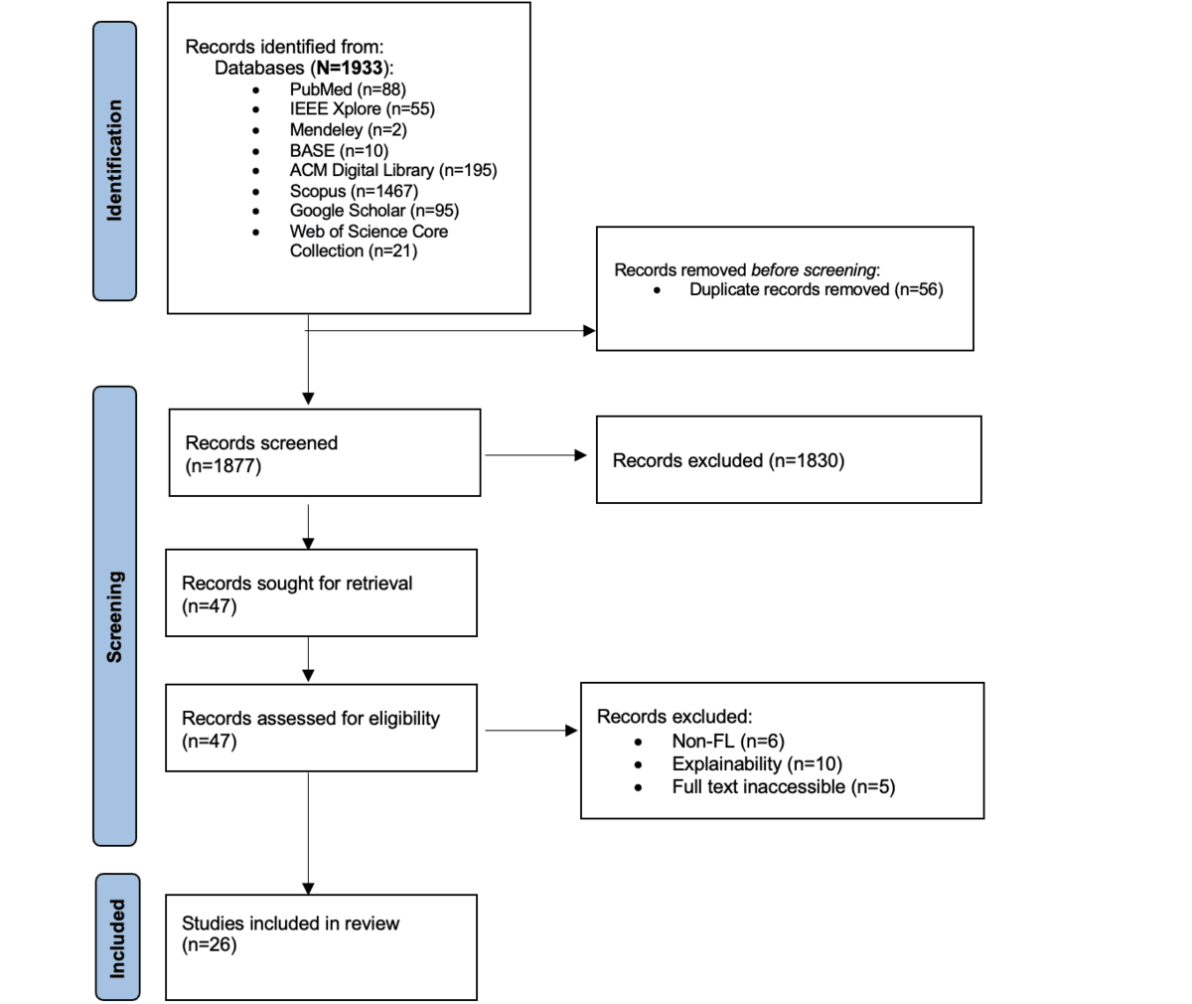
PRISMA flowchart for selection of systematic review literature. FL: federated learning.

This study followed established guidelines for systematic literature review studies [26] and adhered to the PRISMA (Preferred Reporting Items for Systematic Reviews and Meta-Analyses) reporting standards (Figure 4) [27]. Its main objective was to assess the development of XAI within FL. To achieve this, the following review questions were formulated.

### Research Questions

To understand the explainable approaches in FL, research questions (RQs) were raised and grouped under 1 of 3 categories.

#### RQ 1: Trends and Contributions

To understand the contributions of the existing literature, three questions were raised: (1) when were the explainable FL studies published? (2) In which countries or regions are the studies or study applications located, or which countries or regions are the authors of the studies affiliated with? (3) What are the main contributions of the studies identified?

#### RQ 2: Application Areas

The application areas for FL, coupled with the application areas for explainability, were explored based on the following questions: (1) what are the application areas of explainable FL models? (2) What types of FL have been applied in the studies? (3) Why was FL adopted in the studies?

#### RQ 3: Model Explainability

The XAI models and their categories were reviewed based on the following questions: (1) which XAI algorithms or models have been applied or used in the studies? (2) What category of XAI do the models or algorithms used in the studies fall under? (3) What data sources or datasets (if available) were used in the development of the models used in the studies?

### Search Strategy

The reported results followed the population, intervention, comparison, and outcome guidelines [28]. The search string generation process is outlined in Multimedia Appendix 1. The generated search string was adapted to the 8 different databases, as outlined in Multimedia Appendix 2.

### Eligibility Criteria

Of the 1933 initial search results, 26 (1.3%) peer-reviewed studies published between 2016 and 2024 were selected. Inclusion was based on relevance to XAI within any FL context. Exclusion criteria included non–English-language papers, non–peer-reviewed studies, and inaccessible full texts and gray literature as they are not easily retrievable [29].

### Screening

Screening was conducted by 2 independent reviewers using the CADIMA software [30]. Initial screening was based on the titles and abstracts, followed by a blind full-text review. Conflicts were resolved through discussion, and a third party was involved when there was lack of consensus. A strong interrater reliability was achieved, with a κ value of 0.74.

### Data Extraction and Synthesis

Key details from the selected studies, such as title, authorship, affiliation, publication year, data used, and answers to the RQs, were extracted and synthesized using Google Sheets. This process was undertaken by 2 reviewers to minimize bias. Multimedia Appendix 3 contains all the data used for analysis and synthesis.

### Quality Assessment

#### Overview

Quality assessment was undertaken by the 2 researchers (TT and BS) as recommended by Xiao and Watson [26]. The criteria used included handling of overfits, missing data, and use of multiple datasets and validation techniques. The evaluation was based on the PRISMA guidelines [27].

#### Risk-of-Bias Analysis: Individual Studies

The risk of bias of the individual studies focused on potential biases of data selection and model training. The criteria used included handling of overfit and underfit, missing data treatment, use of multiple datasets, and ML evaluation metrics. A total of 69% (18/26) of the studies reported clear mechanisms for mitigating against overfitting and underfitting. In total, 31 (8/26) of the studies lacked evidence of such mitigation. A total of 77% (20/26) of the studies did not address missing data treatment, increasing the risk of data and selection biases [31], especially as most of the studies used preexisting datasets.

**Figure 5:**
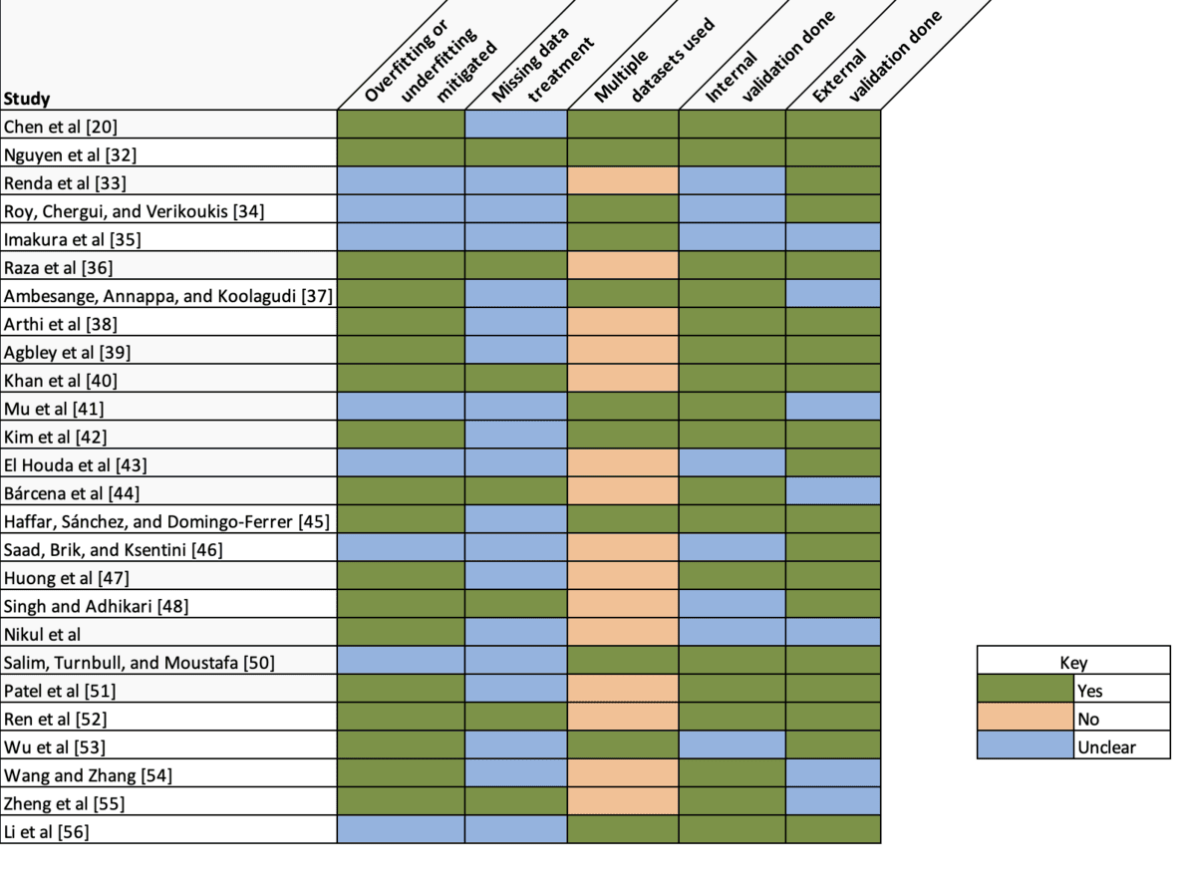
Heat map showing risk mitigation by study for the selected studies.

Figure 5 [20,32-56] shows the risk of bias per study, highlighting how each implemented underfitting and overfitting, missing data treatment, use of multiple datasets, and internal and external validation. Missing data treatment was not clearly identified in most studies (19/26, 73%), with only 27% (7/26) reporting any treatment done. Internal and external validation was conducted in most of the studies (19/26, 73%).

All studies used ML evaluation techniques such as precision, recall, accuracy, *F*_1_-score, mean squared error, mean absolute error, *R*^2^, area under the receiver operating characteristic curve, and the Kolmogorov-Smirnov test. A total of 69% (18/26) of the studies used internal validation techniques (train-test validation split or k-fold cross-validation), with 31% (8/26) of the studies reporting no clear internal validation. Most of the studies (15/26, 58%) had a low risk of bias for their model training, although the lack of missing data training was a key concern.

#### Risk-of-Bias Analysis Across Studies

**Figure 6:**
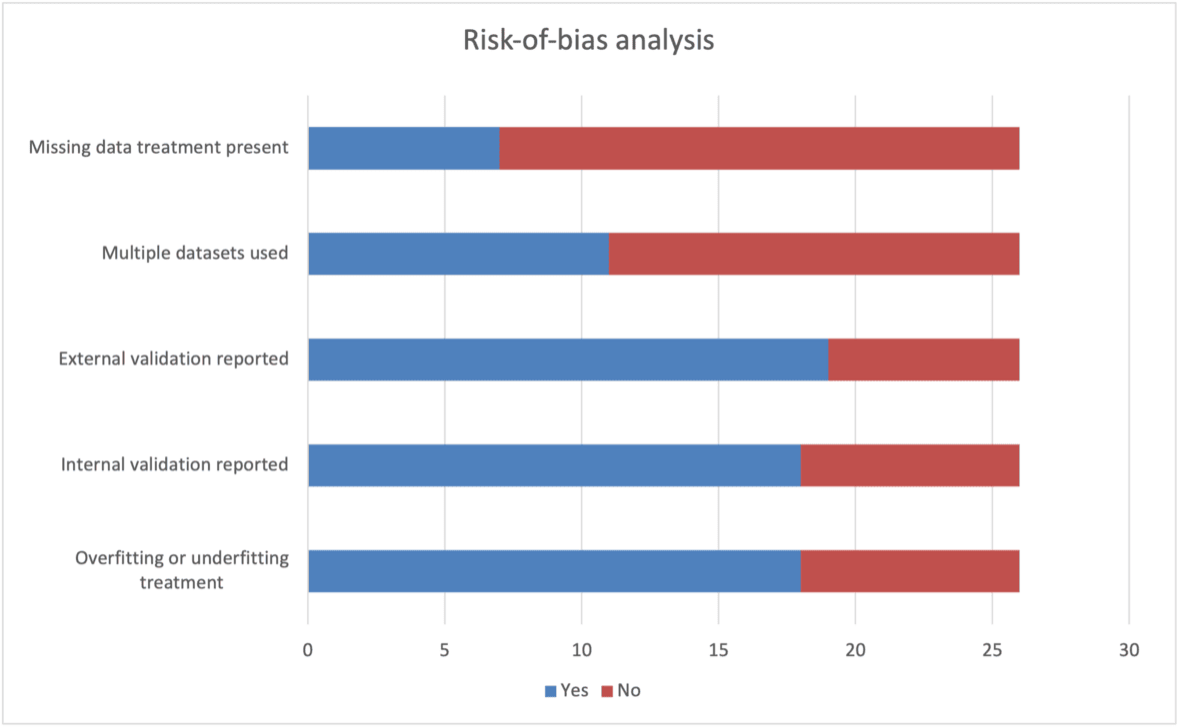
Risk-of-bias analysis showing different bias evaluation methods.

The risk of bias across studies was evaluated on the use of multiple datasets and the use of external ML validation techniques such as benchmarking against state-of-the-art models. A total of 73% (19/26) of the studies performed external validation. In total, 27% (7/26) of the studies lacked external validation. Only 42% (11/26) of the studies used multiple datasets, increasing the risk of bias (Figure 6).

## Results

The selection of the articles is illustrated in Figure 4. The results regarding the RQs are presented in the following sections (Multimedia Appendix 4).

### RQ Category 1: Trends and Contributions

**Figure 7:**
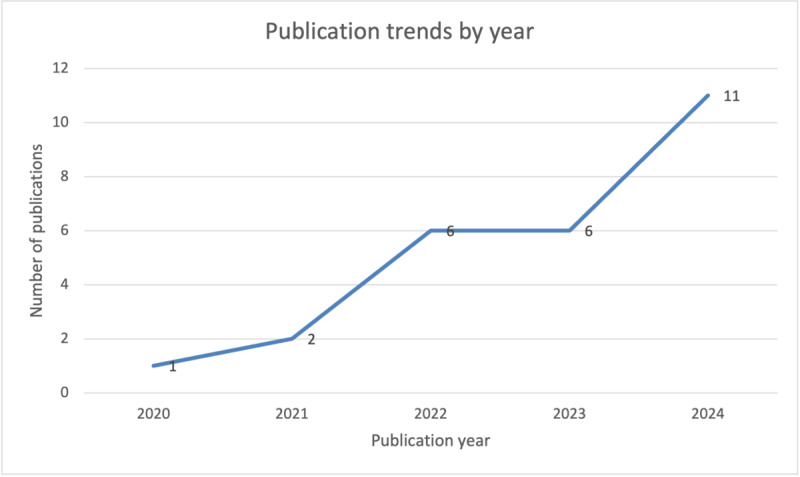
Publication trends for the selected studies by year.

We analyzed the publication trends in explainable FL. While FL emerged in 2016, the first article on XAI for FL was published in 2020(1 publication). The number of articles showed consistent annual growth, culminating in 11 studies in 2024 (Figure 7), which represents the current peak and nearly half (11/26, 42%) of the included studies. The trajectory showed increased interest in this research area despite the low number of total publications (N=26 studies), indicating significant opportunities for future research.

**Figure 8:**
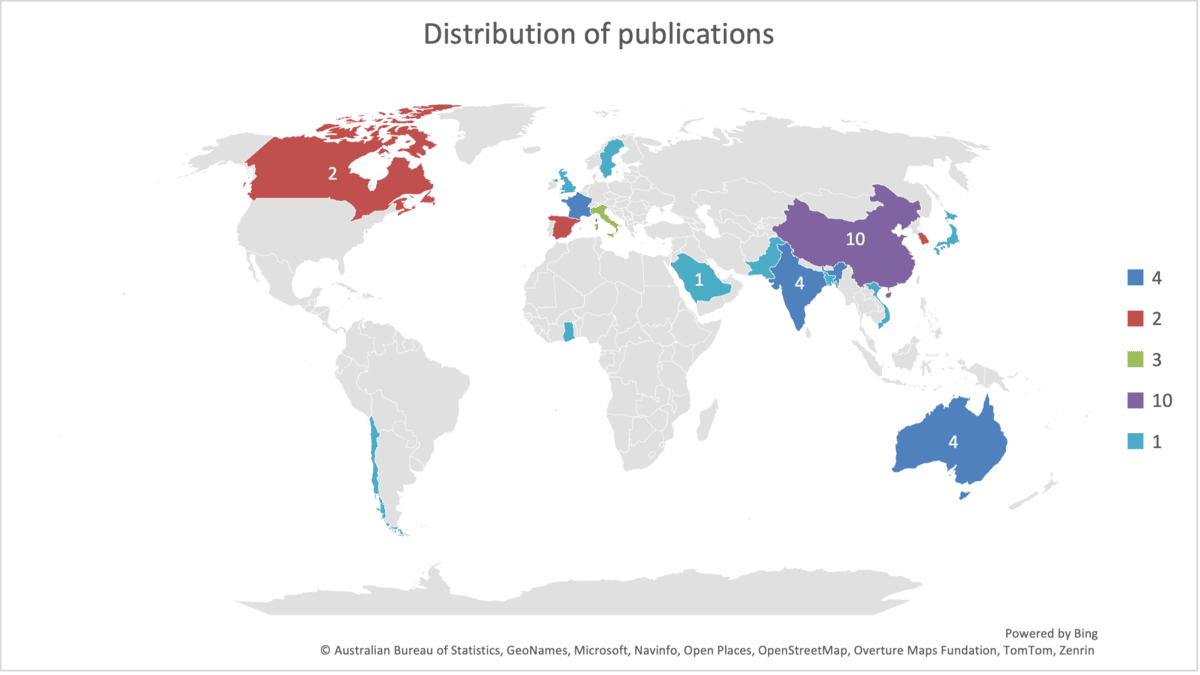
Author affiliation by country for the selected studies (created using the Bing Maps integration in Microsoft Excel [57], which is published under limited license per the Microsoft Bing Maps Terms of Use [58]).

Our analysis of author affiliation revealed a pronounced geographical imbalance, with Asian and European institutions dominating. In contrast, African and South American institutions remained significantly underrepresented, a critical gap given Africa’s potential to benefit from privacy-preserving ML solutions amidst resource constraints. Figure 8 shows the authors affiliation by continent were Asia (23), Europe (11), Australia (4), North America (1), South America (1) and Africa (1).

Despite the African continent having huge potential for rich, diverse, and high-volume data that can be used in ML research, collating and accessing the distributed data (stored in geographically sparse locations or in different institutions, and also in different formats) still poses a challenge. Lack of a computing backbone—including internet connectivity and cloud computing—further leads to data being sourced from high-income countries [59]. Moreover, data scarcity and the lack of proper infrastructure have been highlighted by Fabila et al [60] and Nieto-Mora et al [61] as limiting the research in data-rich diverse areas such as Africa.

Two dominant approaches for achieving explainability in FL systems emerged: those that are intrinsically explainable (ante hoc) [20,32-35] and those that use a surrogate model for explainability (post hoc) [36-53]. In total, 8% (2/26) of the studies [54,55] could not be properly categorized and were classified as “Unspecified.”

### RQ Category 2: Application Areas

#### Overview

**Figure 9:**
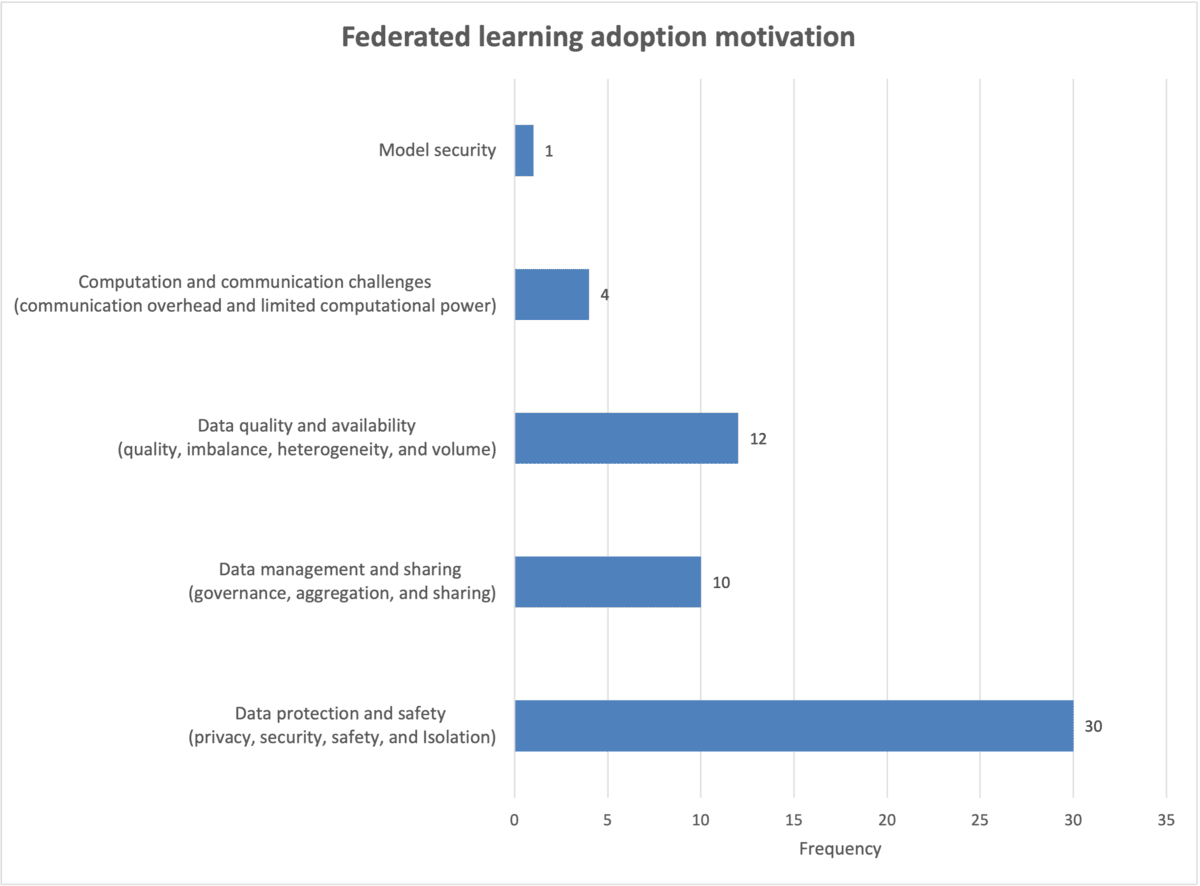
Frequency of federated learning adoption motivations.

The motivations for adoption of FL were analyzed. They were categorized into model security, computation and communication challenges, data quality and availability, data management and sharing, and data protection and safety. The results are shown in Figure 9. The main motivation was data management and sharing, followed by data quality and availability.

#### Application Area and Type of FL Used

The application area and type of FL applied were assessed, and the results are summarized in Table 1. The application area with the highest number of studies was health with 27% (7/26). Networking and finance followed closely with 23% (6/26) and 15% (4/26) of the studies, respectively. Fault detection encompassed 8% (2/26) of the studies, and agriculture, space exploration, urban planning, and social media encompassed 4% (1/26) of the studies each.

**Table 1:** Summary of the studies based on application area, type, and category of federated learning (FL).

Application area and type of FL			Centralized FL		Studies
**Health**					
	Transfer learning	Yes		[36,37]	
	Horizontal FL	Yes		[32,38-40]	
	Vertical FL	Yes		[41]	
**Space exploration**					
	Horizontal FL	—^a^		[42]	
**Networking**					
	Horizontal FL	Yes		[33,43-46]	
	Vertical FL	Yes		[34]	
**Finance**					
	Vertical FL	Yes		[20,42,55]	
	Horizontal FL	Yes		[35]	
**Fault detection**					
	Horizontal FL	Yes		[47,54]	
**Agriculture**					
	Horizontal FL	Yes		[48]	
**Urban planning**					
	Vertical FL	Yes		[49]	
**Social media**					
	Horizontal FL	No		[50]	
**Manufacturing**					
	Horizontal FL	Yes		[51]	
**Energy**					
	Horizontal FL	Yes		[52]	
**Generic**					
	Vertical FL	Yes		[53,56]	

^a^Not applicable.

HFL (17/26, 65% of the studies) was the major type of FL used, with VFL and TL reported in 31% (8/26) and 8% (2/26) of the studies, respectively.

### RQ Category 3: Model Explainability

The selected studies were reviewed for their approach to model explainability, which is essential to building trust in predictions. In FL, understanding model outputs helps assess their reliability and identify the need for adjustments or improvements.

#### XAI Techniques

##### Overview

XAI, first introduced by the Defense Advanced Research Projects Agency in 2015, helps experts understand how ML models arrive at their decisions, thereby increasing trust in the outputs. XAI techniques can be categorized as either global or local depending on the level of explainability. Global XAI techniques offer a broad view of the model’s behavior by highlighting important features. Local XAI techniques focus on explaining individual predictions.

XAI techniques also differ based on whether they are intrinsic to the model (ante hoc or white box), such as decision trees, or applied after training (post hoc), such as LIME [7], which uses simpler models to explain complex ones.

Additionally, some model explainers are model agnostic and can be applied to a wide group of ML models, whereas others are model specific and tailored to particular algorithms, offering deeper insights but requiring more expertise. We provide a brief overview of the techniques in the following sections.

##### LIME Technique

LIME [7] is a popular model-agnostic explainer that uses a simple surrogate model, typically a sparse linear model, trained on locally perturbed data to approximate and explain the individual predictions of a complex model. While it is widely adopted, LIME’s effectiveness depends on the quality of the surrogate fit, and its sampling process introduces uncertainty, resulting in nondeterministic and potentially inconsistent explanations for the same input [62].

##### SHAP Technique

SHAP [8] is a local and global explainer that is based on game theory. SHAP explains a prediction of each instance by computing the contribution of each feature to the prediction. SHAP uses additive contribution to compute a fair value for each feature by computing the contribution of each feature to the final model outcome to understand the importance of each feature. The SHAP explanation is shown in the following equation, where *g* is the explanation model, *x’* is the coalition vector, *M* is the maximum coalition size, and is the feature attribution for feature *i*:




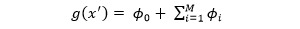




##### Gradient-Weighted Class Activation Mapping

Gradient-Weighted Class Activation Mapping [63] is an explainer that uses the spatial information naturally retained in the last convolutional layer. This is a model-agnostic post hoc explainer that works with different classes of convolutional neural networks. It is a visualization technique that generates heat maps that highlight the important regions of the image that contribute to the model’s prediction.

##### RuleFit

The RuleFit algorithm is a method to generate a model that combines rules and linear regression. First posited by Friedman and Popescu [64] in 2008, RuleFit develops interpretable models that can predict an outcome based on various features. A set of rules is generated from a dataset and then fit into a model using the L1-regularized (least absolute shrinkage and selection operator) regression. The simpler linear models are interpretable like “normal” linear models [65].

##### Partial Dependence Plot

Partial dependence plot (PDP) [66] is an explainer that shows the marginal effect of 1 or 2 features on the predicted outcome of an ML model. It is a post hoc model-agnostic explainer. One or 2 features are selected, and their changes are mapped by changing the values to see their impact on the predicted outcome. The PDP highlights the relationship between the target and the feature as linear, monotonic, or more complex [65]. A newer variant of PDP is called incremental PDP [67], which expands the working of PDP by considering time-dependent effects in nonstationary learning environments. This newer approach considers how the model’s reasoning changes over time while considering the effects of concept drift.

##### Integrated Gradients

Integrated gradients [68] is an axiomatic-based local explainer that attributes the importance value of each input feature of an ML model based on the gradients of the model outputs with reference to the input.

##### Causal Models

Causal models [69] use counterfactual reasoning to explain the cause-effect explanations of a particular model. A counterfactual explanation for a prediction is a description of the smallest change to an input feature that will alter the prediction to a predefined output [65]. Counterfactual explanations describe the causes in the form of “if X had not occurred, then Y would not be the result.” The computation of counterfactual explanations is done by comparing the causal chain paths of the actions not taken by the model [62].

##### Anchors

Anchors [70] are a model-agnostic way of explaining the workings of complex (black-box) models through the use of high-precision rules. Anchors use perturbations to generate the local explanations, but instead of using surrogate models, the explanations are provided using if-then rules that are easy to understand. The if-then rules are called anchors. A rule “anchors” the prediction if changes in the other feature values do not alter the prediction made [65].

##### Deep Taylor Decomposition

Deep Taylor decomposition [71] is an approach for explaining neural networks by decomposing the output of a model into contributions from individual input features. It redistributes the output to the input variables layer by layer. The approach relies on Taylor expansion to determine the relative contributions of the layers. The final relevance scores at the input layer reveal which input features were the most influential in the prediction.

##### Layerwise Relevance Propagation

Layerwise relevance propagation (LRP) [72] is a technique for explaining predictions made by neural network models. LRP identifies the input features that contributed the most to the decision made by the model. LRP relies on deep Taylor decomposition and works by tracing the prediction backward through the network using backward propagation while assigning relevance scores to each input feature [62].

##### Prediction Difference Analysis

Prediction difference analysis [73] generates explanations for neural networks by comparing the model’s prediction when a specific feature is present with the prediction of the model when that feature is absent. The comparison allows for measurement of the feature’s impact on the final model’s prediction. Each feature is removed (knocked out), and a relevance score is assigned to them based on their impact [62].

##### Testing With Concept Activation Vectors

Testing with concept activation vectors [74] is an approach to generate global explanations for neural networks based on the idea of concept activation vectors. It measures the importance of a concept to a prediction based on the directional sensitivity of a concept in the neural network layers. The concept can be anything from color and objects to ideas [65].

##### Explainable Graph Neural Networks

Explainable graph neural networks [75] are model-level explainers that show how graph neural networks make decisions. Explainable graph neural networks use reinforcement learning to build a new graph stepwise, which the original graph neural network can classify as a certain label, for example, “spam.” The new (generated) graph acts as an example for what the model has learned.

#### Explainable FL

XAI can be applied to FL environments to explain the workings of ML models.

##### Explainable FL Techniques Used

This study aimed to explore the types of XAI models used in FL (first question in RQ category 3) and their classification (second question in RQ category 3). Most studies (19/26, 73%) applied existing XAI techniques, especially those originally developed for centralized ML such as LIME [7] and SHAP [8]. A few novel methods such as vertical decision tree ensembles [20] were specifically developed for federated settings. Most reviewed studies (23/26, 89%) used post hoc explainability methods, followed by intrinsically explainable models (5/26, 19%). In total, 8% (2/26) of the studies could not be categorized. Most of the techniques were model agnostic, highlighting the adaptability and widespread use of tools such as LIME in FL environments. Table 2 summarizes the various categorizations of XAI approaches as applied in FL.

**Table 2:** Summary of categorization of explainable artificial intelligence approaches in federated learning, application areas, and performance metrics used.

Approach and model or algorithm		Type (model agnostic or model specific)	Studies	Application area	Performance metrics
**Post hoc**					
	Grad-CAM^a^	Model agnostic	[36,37,39,51,56]	Health care [36,37,39], manufacturing [51], and generic [56]	Accuracy (all studies), precision [36], recall [36,39], and *F*_1_-score [36,39]
	Falcon-INP^b^	Model agnostic	[53]	Generic	Accuracy, precision, and MSE^c^
	RuleFit	Model agnostic	[43,46]	Networking	Accuracy, *F*_1_-score [43], and PDP^d^ and percentage of feature impact [46]
	SHAP^e^	Model agnostic	[43,46-50,52,54]	Networking [50], fault detection [47], agriculture [48], urban planning [49], social media [50], and energy [52]	Accuracy [43,47,49,50,52,54], *F*_1_-score [43,47,50], PDP [46], precision [47,50], recall [47,50], RMSE^f^ [48], MAE^g^ [48], and loss [49]
	LIME^h^	Model agnostic	[38,40,46,49,51]	Health care [38,40], networking [46], urban planning [49], and manufacturing [51]	Accuracy [38,40,49,51], *F*_1_-score [38,40], precision [38,40], recall [38,40], and PDP [46]
	PDP	Model agnostic	[46]	Networking	—^i^
	Causal models	Model agnostic	[41]	Health care	Accuracy
	CPA^j^ Net	Model specific	[42]	Space exploration	Maximum input sensitivity analysis
	Random decision forest	Model agnostic	[45]	Networking	Accuracy
	Rule based	Unspecified	[44]	Networking	MSE and *R*^2^
**Ante hoc**					
	Vertical decision tree ensembles	Model specific	[20]	Finance	AUC^k^ and KS^l^ curve analysis
	Decision trees	Model specific	[33,35]	Networking [33] and finance [35]	MSE, MAE and *R*^2^ [33], and accuracy [35]
	Integrated gradients	Model agnostic	[32,34]	Health care [32] and networking [34]	AUROC^m^ [32], AUPRC^n^ [32], and MSE [34]
**Unspecified**					
	Gradient-based method	Unspecified	[55]	Finance	ROC^o^ and KS curve analysis
	Interpretable adaptive sparse-depth networks	Unspecified	[54]	Fault detection	Accuracy

^a^Grad-CAM: Gradient-Weighted Class Activation Mapping.

^b^Falcon-INP: Falcon Interpretability Framework.

^c^MSE: mean squared error.

^d^PDP: partial dependence plot.

^e^SHAP: Shapley Additive Explanations.

^f^RMSE: root mean square error.

^g^MAE: mean absolute error.

^h^LIME: linear interpretable model-agnostic explanations.

^i^Not applicable.

^j^CPA: Cascading Pyramid Attention.

^k^AUC: area under the curve.

^l^KS: Kolmogorov-Smirnov.

^m^AUROC: area under the receiver operating characteristic curve.

^n^AUPRC: area under the precision-recall curve.

^o^ROC: receiver operating characteristic.

##### Challenges Faced in Explainable FL

Explaining ML models in an FL environment presents unique challenges typically not encountered in centralized setups, especially in real-world scenarios. The challenges include data heterogeneity, security and privacy, communication costs and resource constraints, and scalability.

###### Data Heterogeneity

In centralized ML, data from multiple sources are combined into a single dataset, allowing explainability models to analyze a unified, consistent data distribution. In contrast, FL involves data from different, often heterogenous sources that follow different distributions, resulting in non–independently and identically distributed (IID) data [76]. Non-IID data are common in FL and are characterized by skewed class distributions and varying data volumes across clients [76]. This variability challenges explainability as the explainer model must handle randomly polled clients with diverse and uneven data, complicating interpretation.

###### Security and Privacy

FL was developed to enable ML model training while preserving data privacy, addressing strict data protection regulations. Unlike centralized ML, where XAI techniques risk data leaks or reverse engineering by requiring access to training data, FL introduces new challenges such as vulnerability to model poisoning [77]. Moreover, applying explainability in federated environments can raise privacy concerns as explanation methods might inadvertently reveal some attributes of the client data.

###### Communication Costs and Resource Constraints

FL involves clients sharing model updates via either a centralized or decentralized approach, necessitating continuous and efficient communication. Additionally, the use of perturbation-based explainers such as SHAP adds overheads on client devices due to complex estimation of Shapley values as well as communication costs when sharing the learned perturbations to the central aggregator [78].

###### Scalability

In non-IID FL setups, randomly polling clients is often ineffective, necessitating smarter client selection strategies that prioritize clients with valuable data for improving the global model [79]. Moreover, increasing the number of clients can lead to communication bottlenecks and strain the aggregation server’s resources due to the growing volume of model updates.

## Discussion

### Summary of Findings

This study aimed to understand the current situation in the XAI field and how it has been applied to the field of FL. This was done through a comprehensive review process of the existing openly accessible primary studies on XAI approaches in federated ML. The role of privacy in the choice of ML model was evident in the studies analyzed. FL has proven to be robust and useful in mitigating privacy concerns to comply with privacy legislation and ensure data integrity within the devices [22].

It is noteworthy that most of the studies (10/26, 39%) did not originate from highly sensitive fields such as health and security, which are arguably fields that could benefit most from explainable federated AI approaches. These fields are traditionally conservative, heavily regulated (eg, HIPAA) [11], and still suffer from trust issues due to the lack of explainability of the models. These fields are highly impactful as the problems defined require complex solutions, which necessitate the use of black-box models. Areas such as health, cybersecurity, finance, education, and autonomous vehicles could invariably benefit from explainable FL as they are heavily reliant on privacy and security. Federated XAI could also be applied in edge devices as this would bring the computation closer to the data source while at the same time enhancing privacy and security [80].

The FTL approach, which can help alleviate the challenge of limited training data [81]—the second reported reason for the use of FL—has also not been used fully. Despite the use of real-world datasets, the implementations assessed largely used the HFL approach, which did not fully account for data heterogeneity [82]. Real-world implementations of these approaches might suffer due to the data and environment not being representative. It would be important for more research to be conducted addressing these challenges.

### Implications

There has been a steady increase in the number of studies in the field of FL and XAI. This increase can be mapped from 2016, when FL was first introduced. However, there is still a lot of room for more research to be conducted. The development of explainable FL models can help unlock great potential in the fields of health and security [2], but caution needs to be taken to ensure that the development is not concentrated in specific regions.

Model explainability using state-of-the-art techniques, whether post hoc or intrinsic in nature, has been proven to work well. Several novel explainability techniques that can work well in FL environments, such as those in the studies by Corcuera Bárcena et al [44] and Wang and Zhang [54], highlight the potential for improvement of existing explainability techniques and approaches and development of more robust novel techniques that can perform better in the federated environments. This also offers fertile research potential for experimentation with more real-world data and techniques such as TL.

More research needs to be conducted to mitigate the challenges faced by explainable FL. There is a need to develop models that are scalable and can operate in real-world FL settings where data are non-IID. There is also a need for robust systems that can operate more efficiently when generating the explanations to make them useful for personalized explainable FL. This would help unlock an even greater potential for trustworthy AI.

### Limitations

This review was limited to 26 studies. The novelty of the 2 areas—XAI and FL—meant that a lot of studies (including most studies from the initial total of 1933 identified in the databases) were not eligible for review. Moreover, the strict requirement for primary research and not review papers, coupled with the need for accessible documents, meant that the papers reviewed were limited in nature.

### Conclusions

This study attempted to analyze the existing landscape and provide an overview of the approaches that could be used in implementing XAI in FL. This review was conducted based on the RQs posited, and 26 studies that fit the criteria were assessed. One of the key findings was that, despite the need for explainability in critical areas, there is limited research that has been conducted. More research in these critical areas needs to be conducted to develop more novel approaches that mitigate the challenges. FL remains a useful approach to model development in cases in which privacy is important and limited data exist. This study highlights the potential areas that can be explored by future researchers.

## Appendix

Multimedia Appendix 1 Search string formulation.

Multimedia Appendix 2 Search string results.

Multimedia Appendix 3 Data extraction.

Multimedia Appendix 4 PRISMA checklist.

## Abbreviations

AI: artificial intelligence
CFL: centralized federated learning
FL: federated learning
FTL: federated transfer learning
HFL: horizontal federated learning
HIPAA: Health Insurance Portability and Accountability Act
IID: independently and identically distributed
LIME: linear interpretable model-agnostic explanations
LRP: layerwise relevance propagation
ML: machine learning
PDP: partial dependence plot
PRISMA: Preferred Reporting Items for Systematic Reviews and Meta-Analyses
RQ: research question
SHAP: Shapley Additive Explanations
TL: transfer learning
VFL: vertical federated learning
XAI: explainable artificial intelligence
