# Supplementary material for: Explainable AI Approaches in Federated Learning: Systematic Review
Source: JMIR AI. 2026 Feb 3;5:e69985. doi: 10.2196/69985 (PMC12914235; doi:10.2196/69985)
Supplement: Multimedia Appendix 1 [file ai_v5i1e69985_app1.docx]

## Appendix 1: Search String Formulation

The following search strings were formulated based on the research questions:

*Problem*: The problem under consideration is the types of machine learning federation used. This led to the search string: (“federated” OR “federated learning” OR “federated machine learning” OR “federated AI” OR “federated ML” OR “federated Artificial Intelligence”).

*Intervention*: This relates to the Explainable AI Algorithms and Models used. The search terms therefore are: (“explainable” OR “interpretable” OR “explainable AI” OR “interpretable AI” OR “explainable machine learning” OR “interpretable machine learning” OR “XAI” OR “explainable artificial intelligence” OR “interpretable artificial intelligence”).

*Comparison:* There were no comparative studies to be reviewed hence there is no comparison.

*Output:* The output for the studies considered were the approaches and methods. The search terms thus considered are: (“methods” OR “methodology” OR “approach*” OR “techniques”).

The search terms were therefore combined using AND to get one general query:

*(“federated” OR “federated learning” OR “federated machine learning” OR “federated AI” OR “federated ML” OR “federated Artificial Intelligence”) AND (“explainable” OR “interpretable” OR “explainable AI” OR “interpretable AI” OR “explainable machine learning” OR “interpretable machine learning” OR “XAI” OR “explainable artificial intelligence” OR “interpretable artificial intelligence”) AND (“methods” OR “methodology” OR “approach*” OR “techniques”).*
