# Supplementary material for: Explainable AI Approaches in Federated Learning: Systematic Review
Source: JMIR AI. 2026 Feb 3;5:e69985. doi: 10.2196/69985 (PMC12914235; doi:10.2196/69985)
Supplement: Multimedia Appendix 2 [file ai_v5i1e69985_app2.docx]

## Appendix 2: Search Results

| Database | Search string | Results |
| --- | --- | --- |
| PubMed | (("federated"[All Fields] OR "federated learning"[All Fields] OR "federated machine learning"[All Fields] OR "federated AI"[All Fields] OR "federated ML"[All Fields]) AND ("explainable"[All Fields] OR "interpretable"[All Fields] OR "explainable AI"[All Fields] OR "interpretable AI"[All Fields] OR "explainable machine learning"[All Fields] OR "interpretable machine learning"[All Fields] OR "XAI"[All Fields] OR "explainable artificial intelligence"[All Fields] OR "interpretable artificial intelligence"[All Fields]) AND ("methods"[All Fields] OR "methodology"[All Fields] OR "approach*"[All Fields] OR "techniques"[All Fields])) AND ((ffrft[Filter]) AND (2016:2023[pdat])) | 88 |
| IEEE Xplore | ((“federated” OR “federated learning” OR “federated machine learning” OR “federated AI” OR “federated ML” OR “federated Artificial Intelligence”) AND (“explainable” OR “interpretable” OR “explainable AI” OR “interpretable AI” OR “explainable machine learning” OR “interpretable machine learning” OR “XAI” OR “explainable artificial intelligence” OR “interpretable artificial intelligence”) AND (“methods” OR “methodology” OR “approach*” OR “techniques”)) | 55 |
| Mendeley | (“federated” OR “federated learning” OR “federated machine learning” OR “federated AI” OR “federated ML” OR “federated Artificial Intelligence”) AND (“explainable” OR “interpretable” OR “explainable AI” OR “interpretable AI” OR “explainable machine learning” OR “interpretable machine learning” OR “XAI” OR “explainable artificial intelligence” OR “interpretable artificial intelligence”) AND (“methods” OR “methodology” OR “approach*” OR “techniques”) | 2 |
| Google Scholar | (“federated” OR “federated learning” OR “federated machine learning” OR “federated AI” OR “federated ML” OR “federated Artificial Intelligence”) AND (“explainable” OR “interpretable” OR “explainable AI” OR “interpretable AI” OR “explainable machine learning” OR “interpretable machine learning” OR “XAI” OR “explainable artificial intelligence” OR “interpretable artificial intelligence”) AND (“methods” OR “methodology” OR “approach*” OR “techniques”) | 95 |
| BASE Search | (“federated” OR “federated learning” OR “federated machine learning” OR “federated AI” OR “federated ML” OR “federated Artificial Intelligence”) AND (“explainable” OR “interpretable” OR “explainable AI” OR “interpretable AI” OR “explainable machine learning” OR “interpretable machine learning” OR “XAI” OR “explainable artificial intelligence” OR “interpretable artificial intelligence”) AND (“methods” OR “methodology” OR “approach*” OR “techniques”) | 10 |
| ACM Digital Library | [[All: "federated"] OR [All: "federated learning"] OR [All: "federated machine learning"] OR [All: "federated ai"] OR [All: "federated ml"]] AND [[All: "explainable"] OR [All: "interpretable"] OR [All: "explainable ai"] OR [All: "interpretable ai"] OR [All: "explainable machine learning"] OR [All: "interpretable machine learning"] OR [All: "xai"] OR [All: "explainable artificial intelligence"] OR [All: "interpretable artificial intelligence"]] AND [[All: "methods"] OR [All: "methodology"] OR [All: "approach*"] OR [All: "techniques"]] AND [E-Publication Date: (01/01/2016 TO 12/31/2023)] | 195 |
| SCOPUS | ( "federated" OR "federated learning" OR "federated machine learning" OR "federated AI" OR "federated ML" OR "federated Artificial Intelligence" ) AND ( "explainable" OR "interpretable" OR "explainable AI" OR "interpretable AI" OR "explainable machine learning" OR "interpretable machine learning" OR "XAI" OR "explainable artificial intelligence" OR "interpretable artificial intelligence" ) AND ( "methods" OR "methodology" OR "approach*" OR "techniques" ) AND PUBYEAR > 2015 AND PUBYEAR < 2024 AND ( LIMIT-TO ( DOCTYPE , "ar" ) OR LIMIT-TO ( DOCTYPE , "re" ) ) AND ( LIMIT-TO ( LANGUAGE , "English" ) ) AND ( LIMIT-TO ( SRCTYPE , "j" ) ) AND ( LIMIT-TO ( PUBSTAGE , "final" ) ) AND ( LIMIT-TO ( OA , "all" ) ) | 1467 |
| Web of Science Core Collection | (“federated” OR “federated learning” OR “federated machine learning” OR “federated AI” OR “federated ML” OR “federated Artificial Intelligence”) AND (“explainable” OR “interpretable” OR “explainable AI” OR “interpretable AI” OR “explainable machine learning” OR “interpretable machine learning” OR “XAI” OR “explainable artificial intelligence” OR “interpretable artificial intelligence”) AND (“methods” OR “methodology” OR “approach*” OR “techniques”) | 21 |
