# Supplementary material for: Explainable AI Approaches in Federated Learning: Systematic Review
Source: JMIR AI. 2026 Feb 3;5:e69985. doi: 10.2196/69985 (PMC12914235; doi:10.2196/69985)
Supplement: Multimedia Appendix 3 [file ai_v5i1e69985_app3.docx]

## Appendix 3: Data Extraction

The data extracted from the selected primary studies can be found in:

https://1drv.ms/x/c/f057e56bed55ff11/EaqRd82wQ_tMo911QgQu2gIBpbuMsLW-IidxYkP6CUbOHw
